# Modulation of pathogen-induced CCL20 secretion from HT-29 human intestinal epithelial cells by commensal bacteria

**DOI:** 10.1186/1471-2172-10-54

**Published:** 2009-10-08

**Authors:** Shomik Sibartie, Ann M O'Hara, Jude Ryan, Áine Fanning, Jim O'Mahony, Shaun O'Neill, Barbara Sheil, Liam O'Mahony, Fergus Shanahan

**Affiliations:** 1 Alimentary Pharmabiotic Centre, University College Cork, National University of Ireland, Cork, Ireland

## Abstract

**Background:**

Human intestinal epithelial cells (IECs) secrete the chemokine CCL20 in response to infection by various enteropathogenic bacteria or exposure to bacterial flagellin. CCL20 recruits immature dendritic cells and lymphocytes to target sites. Here we investigated IEC responses to various pathogenic and commensal bacteria as well as the modulatory effects of commensal bacteria on pathogen-induced CCL20 secretion. HT-29 human IECs were incubated with commensal bacteria (*Bifidobacterium infantis *or *Lactobacillus salivarius*), or with *Salmonella typhimurium*, its flagellin, *Clostridium difficile, Mycobacterium paratuberculosis*, or *Mycobacterium smegmatis *for varying times. In some studies, HT-29 cells were pre-treated with a commensal strain for 2 hr prior to infection or flagellin stimulation. CCL20 and interleukin (IL)-8 secretion and nuclear factor (NF)-κB activation were measured using enzyme-linked immunosorbent assays.

**Results:**

Compared to untreated cells, *S. typhimurium, C. difficile, M. paratuberculosis*, and flagellin activated NF-κB and stimulated significant secretion of CCL20 and IL-8 by HT-29 cells. Conversely, *B. infantis, L. salivarius *or *M. smegmatis *did not activate NF-κB or augment CCL20 or IL-8 production. Treatment with *B. infantis*, but not *L. salivarius*, dose-dependently inhibited the baseline secretion of CCL20. In cells pre-treated with *B. infantis, C. difficile-, S. typhimurium-*, and flagellin-induced CCL20 were significantly attenuated. *B. infantis *did not limit *M. Paratuberculosis-*induced CCL20 secretion.

**Conclusion:**

This study is the first to demonstrate that a commensal strain can attenuate CCL20 secretion in HT-29 IECs. Collectively, the data indicate that *M. paratuberculosis *may mediate mucosal damage and that *B. infantis *can exert immunomodulatory effects on IECs that mediate host responses to flagellin and flagellated enteric pathogens.

## Background

Intestinal epithelial cells (IECs) play a crucial role in the maintenance of mucosal homeostasis, and actively sample commensal bacteria, pathogens, and other antigens [1,2]. Under normal circumstances, the mucosal immune system exhibits a restrained response to commensal bacteria whilst retaining an ability to mount appropriate immune responses to pathogenic bacteria. IECs can trigger innate immune responses that activate pro-inflammatory signalling pathways, as well as direct the migration of various effector cells involved in adaptive immunity [2]. Bone marrow-derived dendritic cells (DCs) in the lamina propria also sample commensal and pathogenic bacteria at mucosal interfaces [3,4]. This appears to be achieved by opening tight junctions and extending dendrites between IECs [5]. Encounter with bacteria or bacterial antigens triggers the functional maturation of DCs leading to the generation of antigen-presenting cells that can activate naïve T cells [6]. Therefore, the recruitment of DCs into the epithelium is a prerequisite for the initiation of adaptive immune responses.

Trafficking of leukocytes and DCs to a specific site is dependent on chemokines [7]. Chemokines bind to and activate members of G protein-coupled seven-transmembrane domain receptors that are differentially expressed on leukocytes [8]. The chemokine CCL20 (also known as macrophage inflammatory protein-3α, liver and activated-regulated chemokine, or Exodus-1) selectively attracts effector and memory T lymphocytes, immature DCs, and naïve B cells that express its specific receptor, CCR6 [9]. CCL20 is expressed constitutively at immunological barriers such as the gastrointestinal tract and the skin. The colonic epithelium has been demonstrated as a major source of CCL20 and epithelial expression of this chemokine is elevated in inflammatory bowel disease [10]. The appendix, tonsils, and skin keratinocytes are also significant producers of CCL20 [11-13]. CCL20 expression in IECs is increased by a variety of inflammatory stimuli such as interleukin (IL)-1β, tumour necrosis factor-α, and enteropathogenic bacteria including species of *Salmonella *and *Listeria *[14-16]. It has been shown that flagellin, the protein subunit of bacterial flagella, rather than lipopolysaccharide or invasion, is the key *Salmonella *virulence factor responsible for the induction of epithelial CCL20 [16]. Recent studies found that CCL20 transcription is upregulated by flagellin-Toll-like receptor 5 (TLR5) signaling in the human intestinal epithelial cell lines: T84 and Caco-2 [16,17]. The constitutive and inducible expression of CCL20 implicates an important role for CCL20 both in gut homeostasis and in mucosal immune responses to stress signals. CCL20 expression is upregulated in a variety of inflammatory disorders including appendicitis, atopic dermatitis, rheumatoid arthritis, and inflammatory bowel diseases such as Crohn's disease [9]. This suggests that mucosal inflammation is associated with altered leukocyte and DC trafficking.

Recent evidence supports the role of commensal bacteria in maintaining immune homeostasis within the gut [18-20]. Several strains of commensal bacteria can functionally modulate the epithelium by attenuating the secretion of the neutrophil-recruiting chemokine IL-8 [19-23]. In this study, we examined CCL20 production in response to individual pathogenic and commensal bacteria and whether commensal bacteria could restrain CCL20 secretion specifically from HT-29 IECs. We demonstrate differential stimulation of CCL20 secretion from HT-29 cells and an attenuation of both baseline and inducible CCL20 secretion by commensal bacteria. Collectively, the data could indicate that certain commensal bacteria could contribute to the maintenance of mucosal homeostasis by restraining exaggerated inflammatory responses to the antigenic burden within the gut.

## Methods

### Bacteria and growth conditions

The commensal strains used in this study, *Bifidobacterium infantis *35624 and *Lactobacillus salivarius *subspecies *salivarius *UCC118 [24,25], have been shown previously to have probiotic properties [23,24,26,27]. The bacteria were stored in 50% glycerol at -70°. Prior to use in experiments, *B. infantis *was cultured anaerobically at 37° in de Man Rogosa Sharpe (MRS) (Merck, Darmstadt, Germany) broth supplemented with 0.05% cysteine (Sigma-Aldrich, St. Louis, MO) for 48 hr, whereas *L. salivarius *was cultured anaerobically at 37° in MRS broth for 18 hr. The stationary-phase bacteria were centrifuged and were resuspended in sterile phosphate-buffered saline (PBS).

The flagellated enteropathogenic bacteria used in this study were gram-negative *Salmonella typhimurium *UK1 and gram-positive *Clostridium difficile *ribotype 001. *S. typhimurium *UK1 (kindly provided by R. Curtiss III, Washington University in St Louis, MO) was stored in 50% glycerol at -70°. For experimental investigations, the bacteria were cultured at 37° in tryptic soy broth (Merck) for 18 hr under aerobic conditions.

Subsequently, the bacteria were centrifuged and resuspended in PBS. *C. difficile *001 (kindly provided by J. Brazier, Anaerobic Reference Laboratory, University Hospital of Wales, Cardiff, UK) is a common cause of nosocomial diarrhoea [28]. It produces exotoxins A and B that are pro-inflammatory and enterotoxic in the human intestine [29,30]. *C. difficile *was cultured at 37° under anaerobic conditions in fastidious anaerobic broth (Lab M, International Diagnostics Group Plc, Bury, UK) for 48 hr, followed by sub-culturing in fresh fastidious anaerobic broth for a further 24 hr. Live *C. difficile *bacteria present in the culture broth were used directly to infect HT-29 monolayers.

Subsequently, cell-free supernatants were harvested by centrifugation at 2500 *g *for 10 min. The supernatant was carefully decanted through a 0.2 μm filter to remove any residual bacteria. The absence of viable bacterial cells in the cell-free supernatant was confirmed by plating aliquots of the cell-free supernatant onto Columbia blood agar (Oxoid, Hampshire, UK).

*Mycobacterium avium *subspecies *paratuberculosis *ATCC43109 (American Type Culture Collection, Manassas, VA) was cultured in Middlebrook 7H9 broth (Difco Laboratories, Detroit, MI) supplemented with 10% Middlebrook oleic acid/albumin/dextrose/catalase (OADC) (Becton Dickinson, Franklin Lakes, NJ), 2 ml/l 80% glycerol and 2 mg/ml mycobactin J (Allied Laboratories Inc., Synbiotics Europe) for 2 weeks at 37° in a shaking aerobic incubator. Subsequently, the bacteria were subcultured for a further month in Middlebrook 7H9 broth supplemented with OADC and mycobactin J. *Mycobacterium smegmatis *mc(2) 155 (University College Cork Culture Collection), a non-pathogenic mycobacteria that has been used frequently as a tool in the study of mycobacterial genetics [31], was grown similarly but without the addition of mycobactin J. Prior to use, the mycobacteria were centrifuged and resuspended in PBS. The viability of the mycobacteria cultures was tested routinely using Ziehl-Nielssen acid-fast staining and culture purity was assessed by streaking serial dilutions of the bacterial suspensions on tryptic soy agar (TSA) (Merck). The appearance of any colonies following 24 hr incubation at 37° indicated contamination.

For all assays, bacteria were used in the stationary-phase of growth and were Gram-stained or streaked on TSA (for mycobacteria species) to confirm purity. Bacterial number was estimated by measuring the absorbance at 600 nm, and relating the absorbance value to a standard curve of colony forming units (CFU) on MRS agar (Merck) (for *B. infantis *and *L. salivarius*), TSA (for *S. typhimurium*), Columbia blood agar (for *C. difficile*), or Middlebrook agar (for *M. paratuberculosis *and *M. smegmatis*).

### Epithelial cell culture

In this study, the HT-29 human colonic epithelial cell line (American Type Culture Collection) was chosen because this IEC has been used extensively by us and other groups investigating epithelial responses to bacteria [21-23,32,33]. HT-29 cells were cultured in modified McCoy's 5A medium (GIBCO-BRL, Grand Island, NY) supplemented with 10% heat-inactivated fetal calf serum (FCS) (Sigma) in the presence or absence of 100 U/ml penicillin G and 100 μg/ml streptomycin (GIBCO-BRL). The cells were routinely propagated in 75-cm2 tissue culture flasks at 37° in a humidified, 5% CO_2 _incubator until they approached 80-90% confluency. Subsequently, the cells were trypsinized and used in experimental investigations as specified below.

### HT-29 cell treatments

For all assays, cell viability was determined by trypan blue exclusion, and a known number of HT-29 cells were seeded into 3.8-cm^2 ^12-well plates and grown to confluence. Time and dose response studies were carried out as part of the optimisation phase of this study (data not shown). HT-29 cell-viability and pH changes were also taken into account. Hence, for experiments, *S. typhimurium *was used at 1 × 10^7 ^CFU/ml (MOI 10) for 6 hr given optimal secretion of chemokines within this time. *M. paratuberculosis *and *M. smegmatis *were used at 1 × 10^7 ^CFU/ml or 1 × 10^8 ^CFU/ml (MOI 10 and 100 respectively) for 6 hr and 12 hr. A longer incubation period resulted in impaired cell viability. *C. difficile *was used at 1 × 10^7 ^CFU/ml (MOI 10) for up to 24 hr. Confluent monolayers were also treated with 1 × 10^5^, 1 × 10^6^, or 1 × 10^7 ^CFU/ml live commensal bacteria *B. infantis *or *L. Salivarius *for 6 hr. Formalin-killed commensal bacteria, prepared as described previously [34], were used to treat confluent HT-29 monolayers at a dose equivalent to 1 × 10^7 ^CFU/ml. Dose response studies were performed to determine the optimal concentration of flagellin to use for stimulation of IECs. Subsequently, HT-29 cells were treated with 0.5 μg/ml purified *S. typhimurium *flagellin (InvivoGen Corp., San Diego, CA), a dose used by us in a previous study [23]. In some experiments, HT-29 cells were pre-treated for 2 hr with or without a known dose of commensal bacteria. Subsequently, the cells were incubated under aerobic conditions with an equivalent dose of *S. typhimurium, C. difficile *culture, an equivalent volume of *C. difficile *cell-free supernatant, or with 1 × 10^8 ^CFU/ml mycobacteria, or were treated with 0.5 μg/ml flagellin for varying times. Modified McCoy's 5A medium supplemented with 10% heat-inactivated fetal calf serum and 100 U/ml penicillin G and 100 μg/ml streptomycin was used. Antibiotics were useful in preventing contamination and maintaining a bacteriostatic effect. The concentration (1 × 10^7 ^CFU/ml) of *S. typhimurium *and *C. difficile *at the end-point of the co-culture system was determined by streaking appropriate agar plates. No bacterial growths, pH changes or impaired cell-viability were recorded.

### CCL20 Enzyme-linked immunosorbent assay

Confluent HT-29 cells grown in antibiotic- and serum-supplemented media were treated for varying times with known doses of bacteria or flagellin. Following treatment, immunoreactive CCL20 protein levels in cell-culture supernatants were quantified using an enzyme-linked immunosorbent assay (ELISA) DuoSet kit (R&D Systems, Minneapolis, MN) according to the manufacturer's protocol.

### IL-8 ELISA

Immunoreactive IL-8 protein levels in cell-culture supernatants of bacteria- or flagellin-treated HT-29 cells were quantified using an ELISA DuoSet kit (R&D Systems) according to the manufacturer's protocol.

### Nuclear factor (NF)-κB p65 transcription factor assay

Confluent HT-29 monolayers were treated for 1 hr with known doses of bacteria or flagellin. Nuclear proteins were extracted using the Active Motif Nuclear Extract kit (Active Motif Europe, Rixensart, Belgium) according to the manufacturer's instructions, and the total protein concentration of the lysates was determined by Bradford assay (Bio-Rad, Hercules, CA). Activation of the nuclear factor (NF)-κB p65 subunit in 5 μg of HT-29 nuclear extracts was determined using an NF-κB p65 ELISA-based transcription factor assay kit (TransAM assay) (Active Motif Europe) according to the manufacturer's protocol. The NF-κB detecting antibody recognizes an epitope on p65 that is accessible only when NF-κB is activated. The positive control Jurkat nuclear extract provided with the kit was used to assess assay specificity.

### Statistics

All data are expressed as mean ± SE. Statistical analyses were performed using unpaired two-tailed Student's *t *tests or analysis of variance (ANOVA). *P *values < 0.05 were considered to be statistically significant, and *n *represents the number of independent experiments performed.

## Results

### HT-29 IECs respond differentially to commensal and pathogenic bacteria

**Figure 1:**
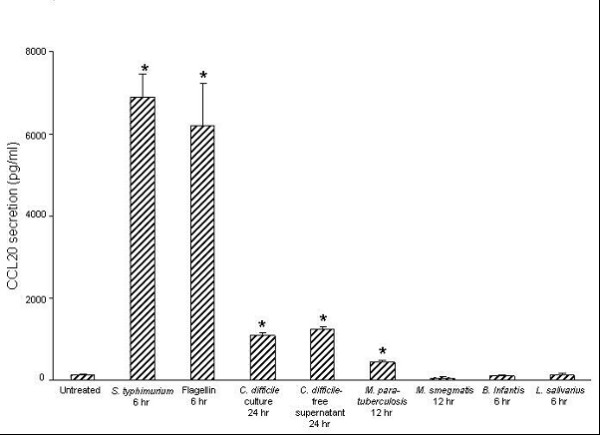
**Intestinal epithelial cells secrete CCL20 differentially in response to various bacteria**. Confluent HT-29 cells were treated with *Salmonella typhimurium *(1 × 10^7 ^colony-forming units (CFU)/ml), flagellin (0.5 μg/ml), *Clostridium difficile *(1 × 10^7 ^CFU/ml), an equal volume of their cell-free culture supernatants, *Mycobacterium paratuberculosis *(1 × 10^8 ^CFU/ml), *M. smegmatis *(1 × 10^8 ^CFU/ml), *Bifidobacterium infantis *(1 × 10^7 ^CFU/ml), or *Lactobacillus salivarius *(1 × 10^7 ^CFU/ml). CCL20 protein levels in cell culture supernatants were measured after 6 hr, 12 hr, or 24 hr as specified on the above graph. Significant levels of CCL20 were only detected after 12 hr incubation with *M. paratuberculosis *and after 24 hr with *C. difficile. M. smegmatis, B. Infantis *and *L. Salivarius *did not result in significant CCL20 secretion at any of the time points or concentrations used. The data are expressed as pg/ml CCL20 and represent the mean ± standard error (*n *= 7 independent experiments). *P < 0.05 relative to untreated cells.

ELISA was used to examine CCL20 and IL-8 protein secretion from confluent HT-29 monolayers treated for 6 hr, 12 hr or 24 hr. HT-29 cells constitutively secreted CCL20, and 119.5 (± 26) pg/ml CCL20 were detected in cell culture supernatants from untreated cells after 6 hr (Fig. 1). Compared to untreated cells, infection with *S. typhimurium *(1 × 10^7 ^CFU/ml) or exposure to its flagellin stimulated significant secretion of CCL20 within 6 hr. The levels of CCL20 induced by *S. typhimurium *or flagellin were comparable. *C. Difficile *significantly stimulated CCL20 secretion only after 24 hr incubation. (Fig. 1). The levels of CCL20 induced by *C. difficile *bacteria (1 × 10^7 ^CFU/ml) (1102 ± (64) pg/ml) and their cell-free supernatants (1232 ± (67) pg/ml) were similar. Dose and time response studies indicated that at a dose of 1 × 10^8 ^CFU/ml *M. paratuberculosis *stimulated significant secretion of CCL20 following 12 hr of infection. *M. paratuberculosis *did not induce CCL20 release at lower doses or at earlier time-points, and *M. smegmatis *did not stimulate CCL20 secretion at any of the doses or time-points tested. Moreover, the commensal bacteria (1 × 10^7 ^CFU/ml) did not induce CCL20 release at any time-point tested (Fig. 1).

**Figure 2:**
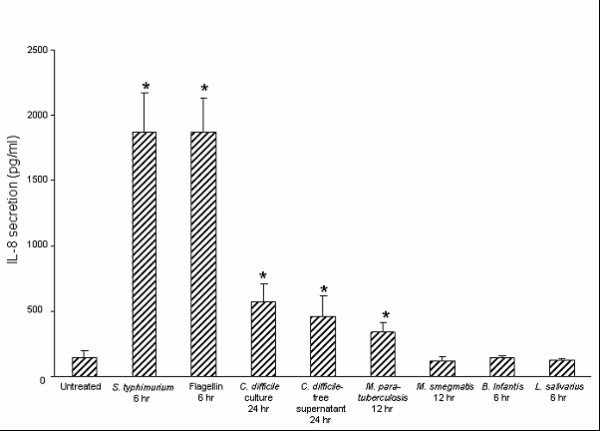
**Intestinal epithelial cells secrete interleukin (IL)-8 differentially in response to various bacteria**. Confluent HT-29 cells were treated with *Salmonella typhimurium *(1 × 10^7 ^colony-forming units (CFU)/ml), flagellin (0.5 μg/ml), *Clostridium difficile *(1 × 10^7 ^CFU/ml), an equal volume of their cell-free culture supernatants, *Mycobacterium paratuberculosis *(1 × 10^8^CFU/ml), *M. smegmatis *(1 × 10^8 ^CFU/ml), *Bifidobacterium infantis *(1 × 10^7 ^CFU/ml), or *Lactobacillus salivarius *(1 × 10^7 ^CFU/ml). IL-8 protein levels in cell culture supernatants were measured after 6 hr, 12 hr, or 24 hr as specified by enzyme-linked immunosorbent assay. The data are expressed as pg/ml IL-8 and represent the mean ± standard error (*n *= 7 independent experiments). *P < 0.05 relative to untreated cells.

Previously, we have shown that flagellin and *S. typhimurium*, but not *B. Infantis *or *L. salivarius*, significantly induce IL-8 protein secretion from HT-29 cells [23]. In the current study, we examined whether *C. difficile *and the mycobacteria species used here stimulate IL-8 secretion from HT-29 IECs. As shown in Fig. 2, infection with *C. difficile *bacteria and their cell-free supernatants significantly stimulated IL-8 protein secretion after 24 hr incubation but not at earlier time points. Exposure to 1 × 10^8 ^CFU/ml *M. paratuberculosis*, but not *M. smegmatis*, significantly stimulated IL-8 protein secretion after 12 hr compared to untreated cells (2.78-fold increase). No significant IL-8 secretion was recorded at earlier time points and at lower doses of *M. paratuberculosis or M. smegmatis*.

### Bacterial-induced NF-κB DNA binding activity in HT-29 IECs

**Figure 3:**
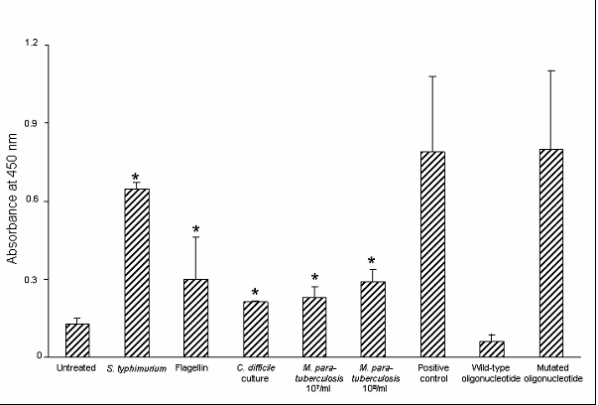
**Bacterial-induced NF-kB DNA binding activity in intestinal epithelial cells**. HT-29 cells were untreated, or were exposed to *Salmonella typhimurium *(1 × 10^7 ^colony forming units (CFU)/ml), flagellin (0.5 μg/ml), *Clostridium difficile *(1 × 10^7 ^CFU/ml), or *Mycobacterium paratuberculosis *(1 × 10^7 ^or 1 × 10^8 ^CFU/ml) for 1 hr. The DNA binding activity of NF-κB p65 in HT-29 nuclear extracts was determined using an enzyme-linked immunosorbent assay-based transcription factor assay. The positive control Jurkat nuclear extract provided with the kit was used to verify assay specificity in competition assays with wild-type or mutated NF-κB oligonucleotides. The data represent the mean absorbance readings ± standard error of five separate experiments. *P *< 0.05 relative to untreated cells.

Both CCL20 and IL-8 are transcriptionally regulated by the transcription factor NF-κB [35,36]. In view of our finding that HT-29 IECs respond differentially to various bacteria, and in particular, that *M. paratuberculosis *triggers CCL20 and IL-8 secretion, we next examined NF-κB activation in bacterial-treated HT-29 cells. Exposure of HT-29 cells for 1 hr to *S. typhimurium *(1 × 10^7 ^CFU/ml), flagellin (0.5 μg/ml), *C. difficile *(1 × 10^7 ^CFU/ml), or *M. paratuberculosis *(1 × 10^7 ^or 1 × 10^8 ^CFU/ml) augmented the DNA binding activity of the p65 subunit of NF-κB compared with untreated cells (Fig. 3). Although the levels of activated NF-κB detected in *C. difficile *and *M. paratuberculosis-*infected cells were less than those detected in *S. typhimurium *infected cells, they were significant nonetheless. NF-κB DNA binding activity was detected in the positive control Jurkat nuclear extract, and the specificity of the NF-κB binding in the assay was confirmed by competition with free wild-type NF-κB consensus oligonucleotide or mutated NF-κB oligonucleotide (Fig. 3). Together the data demonstrate that HT-29 IECs respond differentially to various antigens and, although *M. paratuberculosis *is not as potent an immune activator as *S. typhimurium*, these mycobacteria do trigger inflammatory responses.

### *B. infantis *inhibits CCL20 secretion at baseline

**Figure 4:**
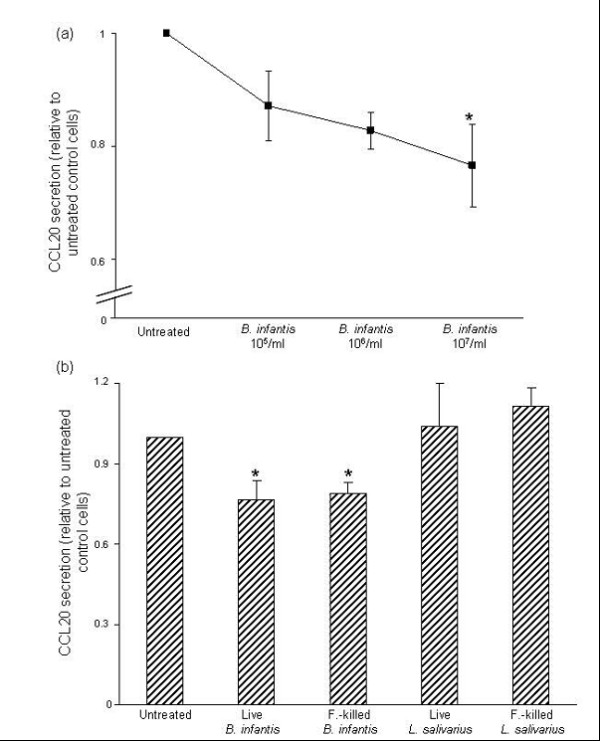
***Bifidobacterium infantis *attenuates the baseline secretion of CCL20 in HT-29 cells**. (a) Confluent HT-29 monolayers were treated with *B. infantis *at doses of 1 × 10^5^, 1 × 10^6^, or 1 × 10^7 ^colony-forming units/ml, and CCL20 levels were measured after 6 hr. *B. infantis *caused a dose-dependent inhibition of baseline CCL20 secretion by confluent HT-29 monolayers. (b) Confluent HT-29 cells were treated with 1 × 10^7^/ml live *B. infantis *or *L. salivarius*, or an equivalent dose of formalin-killed bacteria. CCL20 levels were measured after 6 hr. Both live and formalin-killed *B. infantis*, but not *L. salivarius*, restrained the baseline secretion of CCL20 by HT-29 cells. The data are expressed as CCL20 protein levels relative to untreated control cells and represent the mean ± standard error (*n *= 5 independent experiments). *P < 0.05 compared with untreated monolayers.

In view of our previous finding that commensal bacteria attenuate IL-8 secretion at baseline [23], we examined whether treatment with increasing doses of *B. infantis *or *L. salivarius *(1 × 10^5^, 1 × 10^6^, or 1 × 10^7 ^CFU/ml) affected the baseline secretion of CCL20 by confluent HT-29 monolayers. CCL20 protein levels were measured after 6 hr. In cells treated with *B. infantis*, a dose-dependent inhibition of baseline CCL20 secretion by confluent HT-29 monolayers was observed (Fig. 4a). At a dose of 1 × 10^7 ^CFU/ml, which under the experimental conditions was equivalent to approximately 10 bacteria per epithelial cell, *B. infantis *significantly inhibited basal CCL20 secretion by 24%. Conversely, treatment with *L. salivarius *did not attenuate baseline CCL20 secretion at any dose tested.

In order to determine whether the attenuation of constitutive CCL20 production was dependent on live *B. infantis*, we examined the effect of formalin-killed bacteria on CCL20 protein levels. Similar to live *B. infantis*, at a dose equivalent to 1 × 10^7 ^CFU/ml formalin-killed *B. infantis *significantly inhibited the baseline secretion of CCL20 (Fig. 4b). In contrast, neither live nor formalin-killed *L. salivarius *affected the constitutive secretion of CCL20. Together these data demonstrate that the ability of commensal bacteria to restrain the baseline secretion of CCL20 is species-specific and does not require live bacteria.

### *B. infantis *attenuates CCL20 induction by flagellated pathogenic bacteria

**Figure 5:**
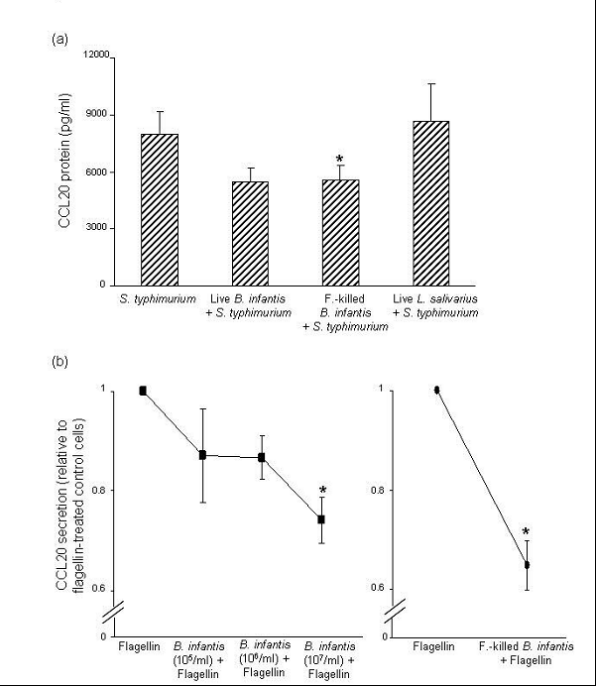
***Bifidobacterium infantis *limits *Salmonella typhimurium- *and flagellin-induced CCL20 secretion**. (a) Confluent HT-29 monolayers were pre-treated for 2 hr with 1 × 10^7^/ml live *B. infantis *or *Lactobacillus salivarius*, or an equivalent dose of formalin-killed *B. infantis *prior to infection with *S. typhimurium*. CCL20 levels were measured 6 hr after infection. Both live and formalin-killed *B. infantis *restrained *S. typhimurium *induced CCL20 secretion (*P < 0.05 compared with *S. typhimurium-*infected HT-29 cells). The data are expressed as pg/ml CCL20 protein and represent the mean ± standard error (*n *= 8 independent experiments). (b) Confluent HT-29 monolayers were pre-treated for 2 hr with live *B. infantis *at doses of 1 × 10^5^, 1 × 10^6^, or 1 × 10^7 ^colony-forming units/ml (left panel) or 1 × 10^7^/ml formalin-killed *B. infantis *(right panel). Subsequently, cells were treated with 0.5 μg/ml flagellin for 6 hr. Pre-treatment with 1 × 10^7 ^live and formalin-killed significantly inhibited flagellin-induced CCL20 secretion (*P < 0.05 relative to flagellin-treated HT-29 cells). The data are expressed as CCL20 protein levels relative to flagellin-treated cells and represent the mean ± standard error (*n *= 7 independent experiments).

As shown in Fig. 5(a), infection of HT-29 monolayers with *S. typhimurium *induced 8006 ± (1140) pg/ml CCL20 after 6 hr. Pre-treatment with live *B. infantis *limited *S. typhimurium-*induced CCL20 secretion (*P *= 0.059), and pre-treatment with formalin-killed *B. infantis *significantly attenuated *S. typhimurium-*induced CCL20 production. The modulation of *S. typhimurium-*stimulated CCL20 production by commensal bacteria was strain-specific; *L. salivarius *did not dampen the IEC CCL20 response to *S. typhimurium*.

Considering that flagellin has been shown to be the key *Salmonella *virulence factor responsible for the induction of epithelial CCL20 [16], we next examined whether *B. infantis *could inhibit IEC responses to *Salmonella *flagellin. Confluent HT-29 monolayers were pre-treated with *B. infantis *(1 × 10^5^, 1 × 10^6^, or 1 × 10^7 ^CFU/ml) for 2 hr, followed by 0.5 μg/ml flagellin for 6 hr. Pre-treatment with *B. infantis *inhibited flagellin-induced CCL20 secretion in a dose-dependent manner. At a dose of 1 × 10^7 ^CFU/ml *B. infantis *this inhibition was significant and was not dependent on the presence of live bacteria (Fig. 5b).

**Figure 6:**
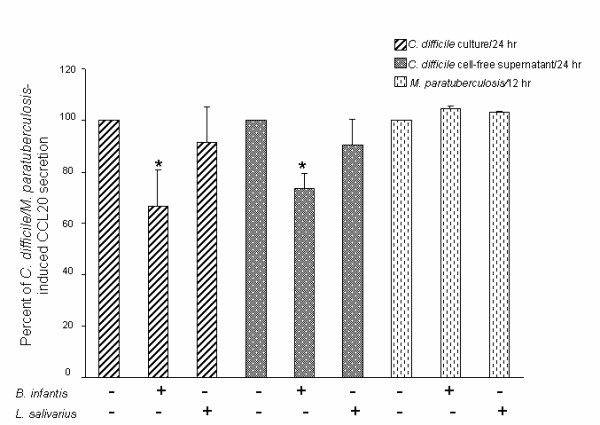
***Bifidobacterium infantis *attenuates *Clostridium difficile*-induced CCL20 secretion**. Confluent HT-29 monolayers were pre-treated for 2 hr with 1 × 10^7 ^colony-forming units/ml (CFU/ml) live *B. infantis *or *Lactobacillus salivarius *prior to infection with 1 × 10^7 ^CFU/ml *C. difficile *bacteria or an equal volume of their cell-free supernatants, or 1 × 10^8 ^CFU/ml *Mycobacterium paratuberculosis*. CCL20 levels were measured 12 hr or 24 hr post-treatment as indicated. *B. infantis*, but not *L. salivarius*, restrained *C. difficile-*induced CCL20 secretion (*P < 0.05 compared with *C. difficile- *treated HT-29 cells). *B. infantis *did not limit *M. paratuberculosis-*induced CCL20 secretion. The data are expressed as per cent of pathogen-induced CCL20 secretion and represent the mean ± standard error (*n *= 6 independent experiments).

We next sought to determine whether *B. infantis *could limit CCL20 induction by pathogenic bacteria other than *Salmonella*. To address this question, we used flagellated *C. difficile *and non-flagellated *M. paratuberculosis*. HT-29 monolayers were pre-treated with live *B. infantis *or *L. salivarius *for 2 hr, followed by treatment with *M. paratuberculosis *for 12 hr or with *C. difficile *for 24 hr. Pre-treatment with *B. infantis*, but not *L. salivarius*, significantly attenuated CCL20 release in response to both *C. difficile *bacteria and their cell-free supernatants by 33.4% or 26.7%, respectively (Fig. 6). In contrast, pre-treatment with the bifidobacteria did not modulate *M. paratuberculosis *induced CCL20 secretion. Collectively, the data indicate that *B. infantis *can restrain CCL20 secretion in response to flagellin as well as to gram-negative and gram-positive flagellated pathogenic bacteria but not to intracellular non-flagellated bacteria.

## Discussion

In the current study, we have demonstrated that HT-29 IECs secrete CCL20 selectively when exposed to various bacterial species. We show that HT-29 IECs release CCL20 in response to *C. difficile, S. typhimurium, M. paratuberculosis*, or bacterial flagellin, but not to *B. infantis*,

*L. salivarius*, or *M. smegmatis*. This study is the first to demonstrate that a commensal strain can attenuate CCL20 secretion at baseline as well as the levels of CCL20 released following exposure to flagellin and flagellated enteropathogenic bacteria. To our knowledge, the present study provides the first evidence that *M. paratuberculosis *can induce the secretion of chemokines and activate NF-κB in HT-29 human IECs. Taken together these data suggest that *M. paratuberculosis *is potentially pathogenic and indicate that *B. infantis *can modulate immune responses to flagellin and flagellated pathogenic bacteria.

*C. difficile *and their toxin-containing supernatants have been shown to stimulate IL-8 production from IECs [37]. However, *C. difficile-*induced CCL20 secretion has not been reported previously. Our finding that HT-29 IECs secrete comparable amounts of CCL20 in response to both *C. difficile *bacteria and their supernatants indicates that components of the supernatant are responsible for the CCL20 release. Flagellin shed from *C. difficile *may be the antigenic factor in the supernatant that elicits the secretion of CCL20. However, the supernatant represents a crude mixture, and the involvement of other components such as exotoxins A or B cannot be excluded. Nevertheless, flagellin from *Salmonella *has been shown specifically to stimulate CCL20 secretion by IECs [16]. This leads to the recruitment of CCR6-expressing immature DCs followed by the uptake of flagellated bacteria and subsequent antigen presentation which initiate adaptive immune responses in the gut. TLR5 activation by flagellin has been shown to stimulate epithelial production of CCL20 and other chemokines like IL-8 [16,17,38]. Flagellin-TLR5 signaling stimulates NF-κB activation to promote CCL20 expression in models of human intestinal epithelial cells [39]. We assume that this mechanism is also responsible for CCL20 expression in our experiments. Recently Muc1 a secreted and membrane bound mucin protein was shown to serve as a receptor that bound *Pseudomonas aeruginosa *and its flagellin, leading to activation of the MAPK pathway [40]. Muc1 is found in abundance in HT-29 cells but further examination is warranted in regards to its role in NF-kB and MAPK signalling [41].

We demonstrate here that *B. infantis *or *L. salivarius *do not elicit CCL20 protein secretion from HT-29 IECs. This extends our previous finding that IECs display immunological quiescence when exposed to these commensal bacteria [23]. In agreement, it has been shown that other commensal bacteria including *Bifidobacterium bifidum, Bacteroides vulgatus*, and *Lactobacillus reuteri *are unable to induce *CCL20 *mRNA expression in IECs [16,42]. However, *Lactobacillus rhamnosus *can augment *CCL20 *mRNA and protein expression in human macrophages, but not in rat uterine epithelial cells [10,43]. Recently, *Lactobacillus rhamnosus GG *(LGG) and *Lactobacillus casei *have been shown not to induce CCL20 or IL-8 from Caco-2 cells. In addition, LGG significantly suppressed the expressions of CCL20 induced by a non-pathogenic flagellated *Escherichia coli *or flagellin when cultured simultaneously [44]. These results are conflicting to our data but reinforce our belief that different strains from the same bacterial species can differ in their molecular interactions with the host.

The mechanism by which *B. infantis *attenuates CCL20 secretion remains to be investigated. CCL20 has been shown to act as an anti-bacterial agent secreted apically as well as basolaterally from IECs. Both secretion apically and basolaterally can be upregulated in response to a number of ligands including muramyl dipeptide, a NOD2 ligand [45]. To survive *B. infantis *could bind or degrade CCL20. The fact that similar effects are observed using dead *B. infantis *renders this hypothesis less likely and we previously demonstrated that only 10% of *B. infantis *remain alive after 2 hr in experimental conditions outlined above [23]. The effects of recombinant CCL20 on *B. infantis *survival should be assessed.

Another mechanistic explanation would be the downregulation of expression of TLR5 or associated molecules in the TLR5 pathway by *B. infantis*. Detailed characterization of the TLR5 pathway for example using RNA interference of TLR5 or downstream MyD88 is warranted and could demonstrate separate pathways of CCL20 induction for *S. typhimurium *and *M. paratuberculosis*.

It could be argued that the suppression of CCL20 by commensal strains could predispose to a suboptimal inflammatory response needed for host defence, especially in the compromised host. The antibacterial properties of CCL20 on pathogens and commensals alike should also be considered. B infantis could negate the beneficial antibacterial properties of CCL20. From our previous observations, *B infantis, L. Salivarius and S. typhimurium *do not survive after 6 hr of incubation in experimental conditions outlined in our study [23]. Hence we believe that *B. infantis *could have more beneficial anti-inflammatory activity than harmful antibacterial-negating effects. This remains to be determined in-vivo. The commensal strain *Bacteroides thetaiotaomicron *has been reported to restrict flagellin-mediated signalling also [19]. The responsible mechanism(s) has not been described, but it is possible that commensal surface structures engage with host cell receptors to modulate inflammatory responses [18,46]. The ability of *B. infantis *and *B. thetaiotaomicron *to restrain the signalling induced by flagellin and pathogenic bacteria may limit exaggerated inflammatory responses to the antigenic burden within the gut and contribute to the maintenance of mucosal homeostasis. Studies by us and others have shown that a variety of commensal bacteria including *B. infantis *and *L. salivarius *can suppress IL-8 secretion at baseline and from infected IECs [21-23]. The anti-inflammatory effects of these commensal bacteria have been shown to be mediated, at least in part, via NF-κB [23]. NF-κB transcriptionally regulates both CCL20 and IL-8 [35,36] and a number of mechanisms by which some commensal bacteria antagonize NF-κB have been described. These include degradation of the NF-κB inhibitor IκB-α, or by the nuclear export of the p65 subunit of NF-κB in a peroxisome proliferator-activated receptor γ-dependent manner [19,20].

Our data shows that *M. paratuberculosis *can activate NF-κB and induce the secretion of IL-8 and CCL20 from HT-29 human IECs. This would suggest that *M. paratuberculosis *may have a role in mediating mucosal damage in the gut. In ruminants and primates, *M. paratuberculosis *causes Johne's disease, a chronic granulomatous enteritis that is very similar to Crohn's disease in humans [47]. Crohn's disease is an immune-mediated inflammatory bowel disorder that appears to be triggered by a complex interaction of environmental, genetic, and immunoregulatory factors [48]. The possibility that *M. paratuberculosis *infection may underlie Crohn's disease has been pursued inconclusively [49,50], but few studies have investigated whether *M. paratuberculosis *can cause mucosal damage in the gut.

## Conclusion

In conclusion, we have demonstrated that *B. infantis *can attenuate CCL20 secretion in HT-29 IECs; thereby modulating responses to limit inflammatory signals induced by flagellin and flagellated pathogenic bacteria. Furthermore, the data demonstrate that *M. paratuberculosis *activates immune responses in HT-29 human IECs. We speculate that *M. paratuberculosis *may mediate mucosal damage and that certain commensal bacteria can contribute to the maintenance of mucosal homeostasis by restraining exaggerated inflammatory response to the antigenic burden within the gut. Similar experiments on different human IEC lines and ex-vivo IECs would further validate our results. Future investigations are expected to improve our understanding of the mechanisms involved in the bacteria-intestinal cell interactions.

## Abbreviations

CCL20: CC-chemokine ligand 20; CFU: colony forming units; DCs: dendritic cells; DMEM: Dulbecco's Modified Eagles medium; ELISA: enzyme-linked immunosorbent assay; FCS: fetal calf serum; IL: interleukin; IECs: intestinal epithelial cells; MRS: de Man Rogosa Sharpe; NF: nuclear factor; NOD/CARD: nucleotide-binding oligomerisation domain/caspase recruitment domain; TLR: toll-like receptor; TSA: tryptic soy agar.

## Authors' contributions

SS carried out work involving mycobacteria, conceived the study and drafted the manuscript. AMOH carried out work with commenseal bacteria and *Salmonella*, conceived the study and drafted the manuscript. JR carried out work involving *Clostridium*. AF carried out immunoassays. JOM and SON were responsible for growing and validating mycobacteria. BS carried out work on commensal bacteria. LOM and FS participated in the design and coordination of the study and helped to draft the manuscript. All authors read and approved the final manuscript.
